# Current Conjugation Methods for Immunosensors

**DOI:** 10.3390/nano8050278

**Published:** 2018-04-26

**Authors:** Zeyang Li, Guan-Yu Chen

**Affiliations:** 1 Department of Chemistry, Massachusetts Institute of Technology, Cambridge, MA 02139, USA; zeyangli@mit.edu; 2 Institute of Biomedical Engineering, College of Electrical and Computer Engineering, National Chiao Tung University, Hsinchu 30010, Taiwan; 3 Department of Biological Science and Technology, College of Biological Science and Technology, National Chiao Tung University, Hsinchu 30010, Taiwan

**Keywords:** immunosensors, polymeric nanomaterials, immobilization methods

## Abstract

Recent advances in the development of immunosensors using polymeric nanomaterials and nanoparticles have enabled a wide range of new functions and applications in diagnostic and prognostic research. One fundamental challenge that all immunosensors must overcome is to provide the specificity of target molecular recognition by immobilizing antibodies, antibody fragments, and/or other peptides or oligonucleotide molecules that are capable of antigen recognition on a compact device surface. This review presents progress in the application of immobilization strategies including the classical adsorption process, affinity attachment, random cross-linking and specific covalent linking. The choice of immobilization methods and its impact on biosensor performance in terms of capture molecule loading, orientation, stability and capture efficiency are also discussed in this review.

## 1. Introduction

Antibody (Ab) and antibody fragment-based biosensors or immunosensors are compact tools capable of providing sensitive and rapid detection or capture of a range of pathogens or cells of interests for further analysis. The history of biosensors dates back to 1956 when Leland C. Clark described the first biosensor which was developed to detect glucose levels in serum samples using a membrane bound biologically sensitive element [1]. Over the past few decades, numerous types of biosensors have been developed for detection of a wide variety of substrates including small molecules, proteins, oligonucleotides, cells, viruses, etc. Immunosensors, a special type of biosensor, frequently applied for the detection of specific antigens or antibodies, are analytic devices that convert the signal generated by antigen-antibody binding events into a measurable signal. To identify new and useful immunosensors, scientists must exploit recent advances in the development of nanomaterial solid supports with ideal surface properties, such as surfaces that employ antigen specific capture molecules for immunochemical reactions or antigen binding. 

The fundamental basis of all immunosensors is to efficiently create stable linkages between desired capture molecules and the nanomaterial. Abs are the most extensively used antigen binding molecules due to their exquisite specificity and affinity. A vast selection of monoclonal Abs (mAbs) have been developed using hybridoma technology to provide excellent tools to detect, perturb, or enhance key components in biological systems. 

Recent efforts have been made to shrink Ab-based tools through the dissociation of full size Abs into smaller antigen binding fragments [2]. Initial truncations were achieved through proteolysis and later by genetic engineering to provide mono- or multi-valent fragments. Such Ab derivatives are bona fide alternatives to full size mAbs as they retain the targeting specificity of the prototype but can be produced more economically with recombinant expression. Examples include antigen-binding (Fab) fragment, single-chain variable fragment (scFv), diabody, minibody, and variable domains derived from heavy chain-only only antibody (VHH) [3,4]. Alternative genetically engineered antibody mimetic proteins, also known as single-scaffold proteins, include designed ankryin repeat proteins (DARPins) [5] and protein A-derived affibody molecules [6]. Together with oligonucleotides-based aptamers, these new alternatives have recently entered immunosensor field [7]. In this review, progress in the development of Abs and Ab-derivative-based biosensors for disease surveillance and monitoring is reviewed, with an emphasis on recent advancement in conjugation methods for the attachment of proteins solid surfaces. The advantages and limitations of antibodies and their derivatives in the context of conjugation methods used in current immunosensors are also discussed. 

## 2. Selection of Antigen Binding Molecules

**Figure 1:**
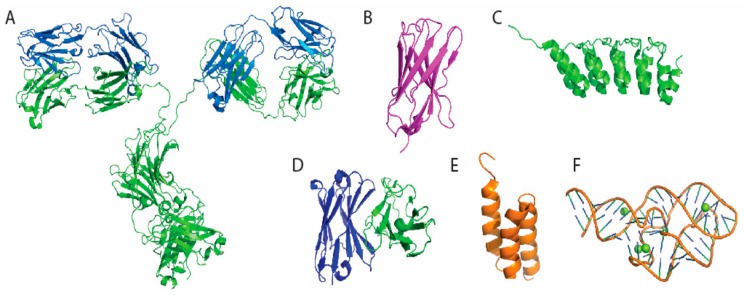
Overall structure of Ab and alternative recombinant binder scaffolds used in biosensors. (**A**) IgG2a monoclonal antibody with two heavy chains colored in green and two light chains colored in blue (PDB:1IGT) [18]; (**B**) The green fluorescent protein (GFP)-VHH (PDB: 3OGO) [19]; (**C**) DARPin against tubulin beta chain (PDB: 4DUI) [20]; (**D**) anti-fluorescein ScFv (PDB: 1X9Q) [21]; (**E**) human epidermal growth factor receptor 2 (HER2) binding affibody (PDB: 2KZJ) [22]; (**F**) 5-hydroxytryptophan aptmer (PDB: 5KPY) [23]. We used PyMol to generate all the structures in this figure.

**Figure 2:**
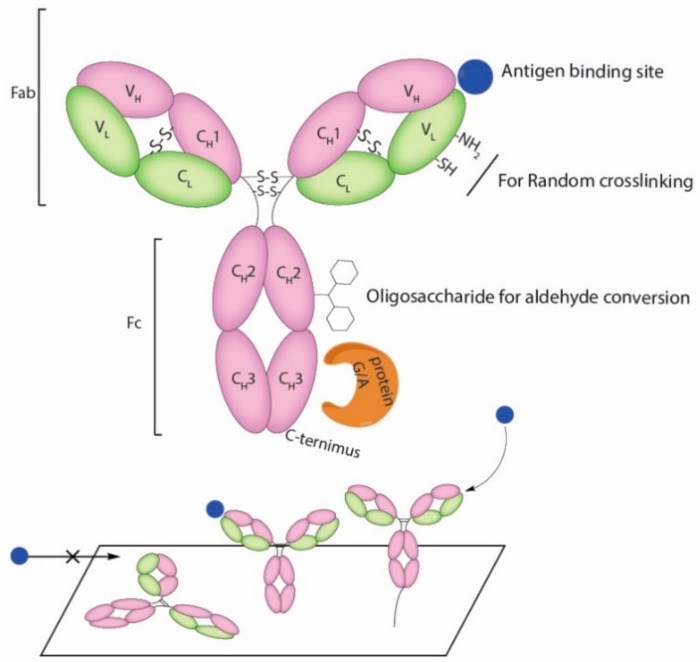
Functional groups on Abs used for conjugation and the result of random and oriented immobilization onto surfaces.

Abs or immunoglobulins (Igs) are highly soluble serum glycoproteins which can be divided into five main isotypes (IgA, IgD, IgE, IgG, and IgM) based on their heavy chain constant region sequences [8]. Polyclonal antibodies (pAb) can be easily raised in animals such as rat, rabbits, goats, and sheep. They are frequently used in immunosensors for pathogen detection. However, multiple epitopes may often be recognized by pAb since their source is a pool of Ab secreting B cells [9]. In cases where highly specific binding is required, monoclonal antibodies (mAb) are more desirable and can be generated through the use of hybridoma technology [10]. Splenocytes from an immunized animal are commonly used as the source of Ab-producing B cells for myeloma fusion. The resulting hybridoma cells are immortal cell lines secreting full size Abs [11]. The pool of hybridomas can be further screened against targeted antigens to identify suitable single cell candidate for monoclonal Ab production. Among the five main isotypes of Ig, IgG is the main type of Ab found in blood and extracellular fluid [8]. The general structure of an IgG is shown in Figure 1A, and it consists of four polypeptide chains, i.e., two heavy chains and two light chains, which are joined together by disulfide bonds. The antigen-binding (Fab) region, composed of one constant and one variable domain from a paired heavy and light chain, provides the site responsible for antigen binding (Figure 2). Conjugation reactions may result in deleterious effect on antibody avidity [12]. Thus, immobilization strategies should be carefully designed so that Fabs are left unaltered throughout the process. High degree random conjugation may inevitably results in changes in antigen binding characteristics and, in more extreme scenario, a complete loss of function [13]. In addition, different immobilization methods may lead to uniform or random orientation of Abs on solid support [14]. The ability to control the site of protein (Ab) modification is essential to avoid the destruction or steric hindrance of immobilized Fabs. In principle, modification sites should be kept far away from the Fabs to achieved the best result.

Although full-size Abs generated through immunization have been widely used for immunosensors since the beginning of biosensor development, recently developed recombinant molecules generated in vitro have many advantages over conventional Abs. The advantages of these alternative scaffolds include their compact sizes, excellent thermal and chemical stabilities, as well as low production costs [4,15]. For example, to obtain useful VHHs found in camelids (Figure 1B), a cDNA library can be obtained from an immunized llama or alpaca to serve as a pool of amplified DNA fragments for generation of the phage display library [15]. Phage that bind target during panning process can then be recovered, sequenced and transferred to a periplasmic expression vector to obtain the final VHH protein from *E. coli* expression. VHH binders with desired properties can be recombinantly expressed with tag sequences, protein fusion partners, or artificial amino acids through covalent modification [16]. Similarly, DARPins (Figure 1C), scFv (Figure 1D), and affibody (Figure 1E) can also be screened from existing libraries as alternative binders. In addition, peptide and oligonucleotide based binding molecules, such as aptamers (Figure 1F), can be screened from a pool of random sequences to acquire molecules with desired binding specificity and affinity [17].

## 3. Immunosensor Types and Common Material Selection

Supporting materials of an immunosenor can be selected based on analytic needs (Table 1). Optical transducers that exploit light absorption, luminescence, and fluorescence often require optically transparent materials as support. Surface plasmon resonance (SPR) sensors rely on the unique optical properties of metallic nanostructures (gold, silver, etc.) [24]. Electrochemical immunosensors produce electrical charges for the quantitative analysis of target molecules. Many novel nanomaterials, such as carbon nanotubes, graphene, indium tin oxide, nanowire, hydrogels, and metallic nanoparticles, are employed to construct a high-performance electrode for signal output [25]. Many attempts have been made to improve the electrochemical properties of supporting material to increase electro-catalytic trait, create excellent electron transfer ability and excellent biocompatibility [25,26]. Piezoelectric immunosensors exploit the piezoelectric effect which occurs in various crystalline substances. The piezoelectric immunosensor is known to be one of the most sensitive analytical instruments with the ability to detect antigens in the picogram range [27,28].

## 4. Current Conjugation Methods

The performance of an immnosensor depends upon three key factors: (1) the binding affinity and specificity of antigen binding molecules; (2) the accessibility and proportion of binding sites intact after immobilization; and (3) the density of binding molecules coated on the surface of immunosensor. Different strategies for immobilization may result in different outcomes and efficiencies (Figure 2). Immobilized Abs can adopt several different orientations depending on the method applied. Adsorption method often results a “flat-on” orientation, with the Fc and Fab fragments lying flat on the surface [37]. This confirmation can result in hindrance of antigen access to antibody binding sites, which eventually lead to the decrease in antigen binding capacity [38]. Specific orientation is always ideal but not as easily achieved as adsorption, since site specific modification of antigen binding molecules commonly require incorporation of a unique reactive group. Affinity based attachment using protein A and protein G provides another solution. These proteins display multiple binding sites specific to the Fc potion of Abs and lead to a predominantly “tail-on” attachment of Abs. Further improvement can be done by introducing a linker between protein A and protein G to the system. The immobilization strategies should be also compatible with the targeted surface materials, such as gold, copper, iron, silicon, hydro-gel, carbon nanotubes, and graphene oxide. We presented the most popular conjugation methods for each different binding molecule category (Table 2).

**Figure 3:**
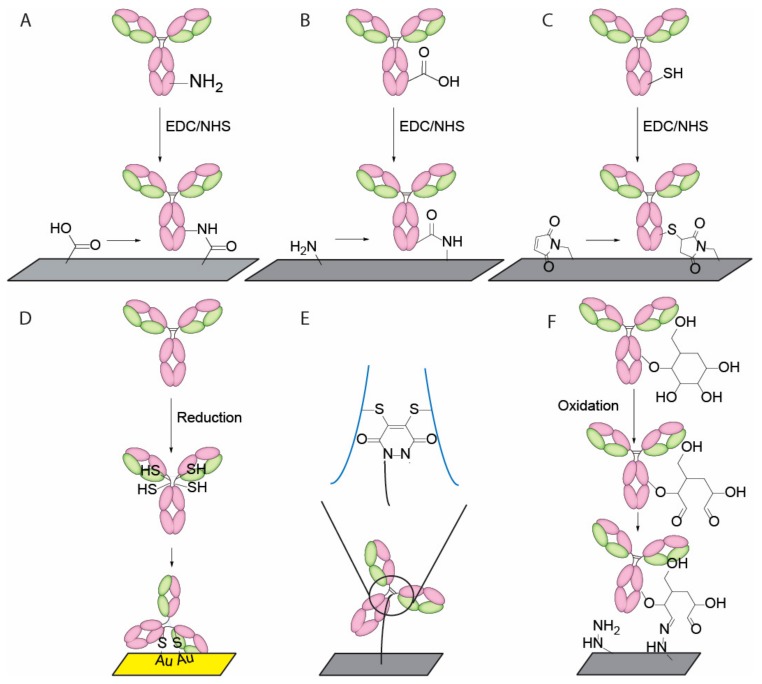
Ab immobilization scheme. (**A**) EDC/NHS coupling of Ab surface amine to carboxyl and (**B**) carboxyl groups to amine groups; (**C**) Sulfhydryl-reactive chemical group coupling to Ab surface thiol groups; (**D**) Reduction of antibody disulfides to reactive thiols for gold substrates binding; (**E**) Reduction of antibody disulfides for site specific pyridazinedone coupling; and (**F**) Oxidation of sugar chains for reactive aldehyde groups.

Physical adsorption of Abs onto hydrophobic surfaces such as polystyrene offers the simplest attachment. However, the process is uncontrollable in terms of orientation and stability, and often results in denaturation and detachment of protein on surface [37]. Protein G and protein A are small bacteria derived moieties bind in a specific orientation to Fc region [45]. This method offers non-covalent but mostly “tail-on” Ab attachment. Random crosslinking methods provide another mode of attachment as amine, carboxyl and thiol groups are abundant throughout the surface of antibody. Amine and carboxyl coupling is commonly achieved by using carbodiimide as a carboxyl activation reagent in combination of succinimidyl ester (NHS) for improved efficiency [46]. This method, also known as EDC/NHS coupling, can be used to robustly create covalent linkage via amide bond formation (Figure 3A,B). Reactive primary amine and carboxyl groups on Ab surface are mostly lysine, aspartic acid and glutamic acid side chains, which are usually abundant in Fab region due to its polar nature. Thus, it is impossible to control the orientation and predict the outcomes of Fab region immobilization as the immobilized product is a mixture of Abs modified at different molar equivalents and positions. Similarly, reactive thiol groups (cysteine side chains) can also be targeted for maleimide or iodoacetamide reaction (Figure 3C). Additionally, disulfide bonds can also be reduced as an alternative source of thiol groups. In addition to the classical maleimide reaction or gold surface attachment (Figure 3D), Baker et al. and Chudasama et al. reported the usage of pyridazinedone as a way to yield a more homogenous product with better retention of structural bond (Figure 3E) [48]. Several other approaches are reported to achieve specific orientations. Kang et al. reported a site specific biotinylation strategy using the sugar moiety on the Fc region (Figure 3F) [49]. Oxidation of sugar chains yield reactive aldehyde groups, which can be used to covalently link Abs in an oriented manner without disturbing the structural integrity. There are several other immobilizations methods reported recently. Bilgicer and co-workers exploited the conserved nucleotide binding site (NBS) on the Fab region to achieve site specific labeling using UV-cross linking method [51]. Boozer and others reported a DNA-directed Ab immobilization method by using ssDNA pre-conjugated to Ab to form a self-assembled monolayer on the surface coated with complementary sequence [52]. Another Ab site-specific labeling strategy is to use formylglycine-generating enzyme (FGE) to install an aldehyde tag on a specific pentapeptide sequence, which may then react with aminooxy-containing surface linkers [66].

**Figure 4:**
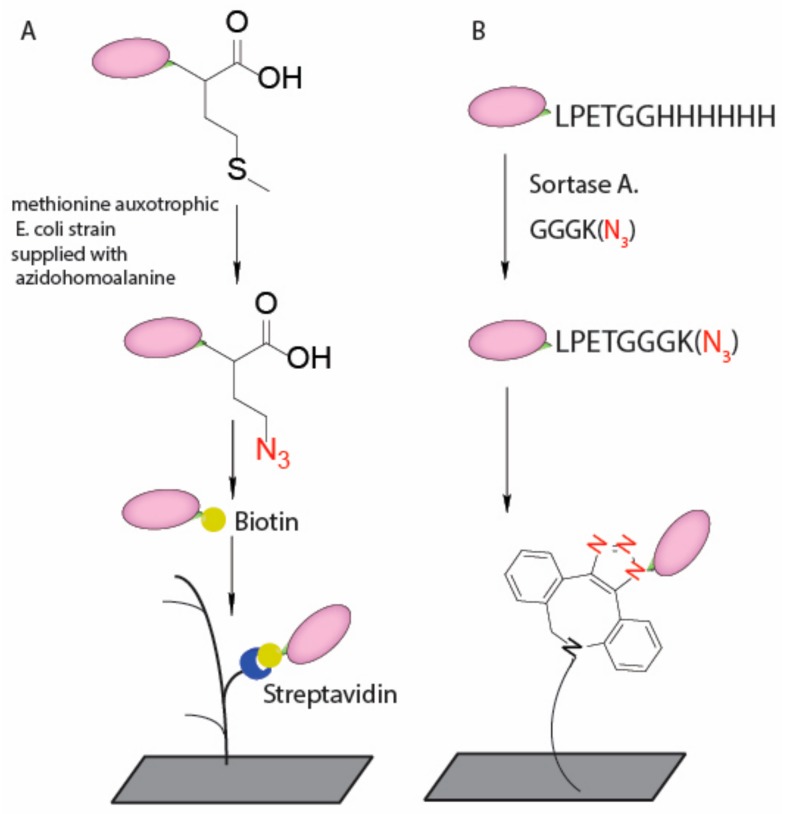
Oriented immobilization scheme for VHHs. (**A**) C-terminal N_3_ group introduced by artificial amino acid incorporation followed by conversion to biotin group for streptavidin binding; and (**B**) C-terminal N_3_ group attached via sortase mediated transpeptidation followed by site specific attachment on DBCO modified surface.

VHHs, also known as nanobodies, are recombinant, antigen-specific, single-domain, variable fragments of camelid heavy chain-only antibodies. Compared to full size IgGs, VHHs can be expressed in high yield in bacterial systems. The small size (~14 kD) provides significant advantages in medical diagnostic and therapeutic applications [67]. However, due to their small size, VHHs have a significantly higher percentage of their surface involved in binding interactions compared to full size IgGs; therefore, site-specific installation of linker is particularly important for immunosensors that use VHHs. Beekwilder et al. reported an oriented labeling method using azide functionalized VHH onto a cyclooctyne-tailored sensor surface (Figure 4A) [53]. They emphasized the importance of oriented immobilization as it increased sensor efficiency up to 800-fold compared to randomly labeling. In addition, transpeptidase can also be used to create site specific modification on VHH since a short peptide recognition sequence can be easily incorporated into recombinant expression vectors. The Ploegh group incorporated the sortase recognition motif, LPXTG, at the C terminus of VHHs, which can then be used for installation of a short GGG peptide with a biorthogonal handle (Figure 4B) [55,56,68,69,70].

**Figure 5:**
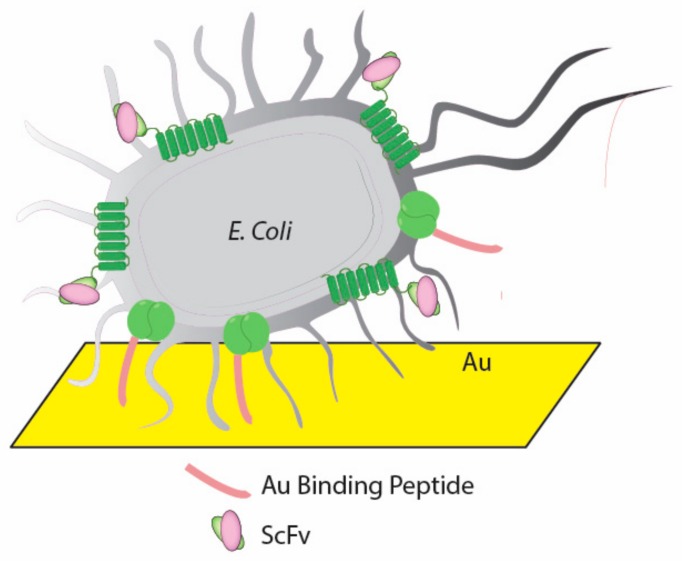
ScFv and gold-binding peptide dual-expression system in *E. coli* [60].

ScFvs are a type of fusion protein which contains variable regions of the heavy (V_H_) and light chains (V_L_) of Abs connected via a short peptide linker. Shen et al. optimized a 15-mer peptide linker (RGRGRGRGRSRGGGS) to increase the adsorption efficiency on anionic charged biosensor surface [57]. Chen et al. developed a cancer marker monitoring platform that constructed by a dual-expression system in *E. coli* which can display anti-cancer ScFvs and gold binding peptide on the surface of bacteria at the same time (Figure 5). In this case, ScFvs are fused at the C-terminus of the extracellular domain of a transmembrane protein (Lpp-OmpA) and achieve a fixed orientation display of ScFv on *E. coli* surface. 

Other novel alternatives, such as DARPins and Aptamers can also serve as the binding moiety in immunosensors. DARPins are a novel class of non-IgG scaffolds based on naturally occurring ankyrin repeats. DARPins are small (13–20 kDa) and highly soluble in aqueous solution. Deyev and others reported that DARPins can bind tightly to gold nanoparticles (GNPs) via adsorption [61]. 

Aptamers are single-stranded DNA or RNA (ssDNA or ssRNA) oligonucleotides or peptides engineered through repeated in vitro selection or equivalent methods. However, aptamers have distinct limitations, especially for those composed of DNA or RNA. The rapid degradation of aptamers by nucleases in biological media is a serious problem. Such degradation causes instability which is unacceptable for biosensor application. Despite such limitations, many successful attempts have been made to create aptamer-based biosensors in relatively nuclease free systems. One approach employed terminally functionalized thiol group for gold surface binding [64]. Other functional groups such as primary amine or activated carboxylic acid are also commonly used for covalent conjugation [65].

## 5. Conclusions

The performance of immunosensors is closely associated to the antigen binding molecules and immobilization approach. While Abs have been increasingly used as detection elements in immunosensors, recent development in antibody derivatives and other alternative binding molecules raise new opportunities and possibilities to create highly stable, efficient, and economically feasible diagnostic device. In this review, a wide range of immobilization strategies are presented for Ab and Ab alternatives and their applications on various nanomaterial surfaces are discussed. Pros and cons of each method are presented. The uniform orientation conferred by site-specific immobilization is essential for small Ab alternatives to retain their binding efficiency. 

## Figures and Tables

**Table 1:** Immunosensor types and common materials used.

Immunosensor Type		Common Materials	References
Optical	Evanescent wave	Quartz, glass, graphene oxide (GO) sheets, hydrogels	[24,29,30,31]
	Surface plasmon resonance (SPR)	Silver, gold, copper, aluminum	
Electrochemical	Conductive	Carbon, indium tin oxide, carbon nanotube, hydrogels, polythiophene	[32,33,34,35]
	Amperometric	Graphite, Lipid, Platinum, Gold, Nickel	
Piezoelectric	Bulk acoustic wave	Aluminium phosphate, aluminium nitride, zinc oxide, crystalized topaz, crystalized tourmaline, barium titanate, gallium orthophosphate, lead titanate	[27,28,36]
	Surface acoustic wave		

**Table 2:** A summary of popular conjugation methods.

Type of Antigen Binding Molecules	Types of Immobilization	Functional Group	Orientation	References
**Antibody**	Adsorption	Various	Random	[39,40,41,42]
	Affinity	Antigen-antibody reaction	Partially oriented	[42,43,44]
		Protein A or G (non-covalent) binding	Partially oriented	[41,45]
	Radom crosslinking	Amine/carboxylic acid	Random	[43,44,46,47]
		Thiol group	Random	[44,46,48]
		Sugar chain on C_H_2	Partially Oriented	[49,50]
	DNA-directed	Nucleotide Binding Site ssDNA hybridization	Uniformly oriented	[51,52]
	C terminus	Enzyme mediated biotinylation	Uniformly oriented	[49]
**VHH**	C terminus	non-natural amino-acid	Uniformly oriented	[53,54]
	C terminus	Enzyme mediated transpeptidation	Uniformly oriented	[55,56]
**scFv**	Tag mediated	Cysteine or Histidine containing linker	Partially Oriented	[57,58,59]
	*E. coli* surface displayed	Genetic fusion	Uniformly oriented	[60]
**DARPins**	Radom crosslinking	Amine group	Random	[61,62]
**Aptamer**	Terminal modification	Thiol	Uniformly oriented	[63,64,65]
